# Breaking the strength limit: Negative-excess-energy interfaces push Ni alloys toward theoretical strength

**DOI:** 10.1016/j.fmre.2026.02.003

**Published:** 2026-02-18

**Authors:** Fenghui Duan, Zhen Yu, Hanzheng Xing, Jian Lu

**Affiliations:** a Laboratory of Nanomaterials & Nanomechanics, Department of Mechanical Engineering, City University of Hong Kong, Hong Kong 999077, China; b Hong Kong Branch of National Precious Metals Material Engineering Research Centre (NPMM), City University of Hong Kong, Hong Kong 999077, China; c Centre for Advanced Structural Materials, City University of Hong Kong Shenzhen Research Institute, Shenzhen 518057, China; d City University of Hong Kong Matter Science Research Institute (Futian), Shenzhen 518045, China

Strengthening metallic materials has long been a central pursuit in materials science. In the 1920s, Frenkel proposed the concept of ideal strength: the theoretical maximum stress that a perfect, defect-free crystal can sustain before fracture [1]. This value typically lies between *E*/30 and *E*/10, where *E* is the elastic modulus. However, the strength of real metals is much lower because of the ubiquitous presence of defects such as dislocations. Over the past century, several strengthening strategies have been developed, including grain boundary or twin boundary (TB) strengthening, solid-solution strengthening, precipitation strengthening, and phase transformation strengthening. All these approaches rely on introducing structural defects to impede dislocation motion. Yet, when defects become overly dense, materials transition from “dislocation-controlled hardening” to “defect-induced softening”. In particular, metals significantly strengthen as their grain size or TB spacing is reduced to the nanometer scale, but soften when the size and spacing fall below ∼10–15 nm [[2], [3], [4]]. Below this “strongest size”, interfaces themselves become unstable and act as active deformation sources through mechanisms such as grain boundary sliding, grain rotation, and detwinning, causing the breakdown of the Hall-Petch relationship.

Now, writing in *Science*, J. X. Li and colleagues report a breakthrough that overcomes this long-standing limitation [5]. By engineering a supersaturated Ni–Mo alloy with a high density of coherent interfaces exhibiting negative excess energy, they demonstrate continuous strengthening at characteristic length scales below 1 nm. Upon crystallizing pulse-electrodeposited amorphous Ni–Mo alloys at 773 K and 973 K, the authors observed the formation of extremely dense planar faults within the grains. High-resolution high-angle annular dark-field scanning transmission electron microscopy (HAADF-STEM) images revealed that these planar faults are formed by alternating stacks of face-centered cubic (FCC) and hexagonal close-packed (HCP) layers (Fig. 1a, b), with the minimum interlayer spacing being only approximately 0.7 nm. The formation of such an extremely high density of coherent interfaces during crystallization is highly unusual. Typically, crystallization from an amorphous state reduces the system’s free energy and produces nearly defect-free grains. In contrast, dense stacking faults and coherent FCC/HCP interfaces emerge spontaneously in this system. This unexpected behavior arises from a subtle interplay among chemical short-range order, atomic diffusion, and local lattice displacement during crystallization. The supersaturation of Mo atoms significantly lowers the stacking fault energy, promoting fault formation and stabilizing mixed FCC/HCP stacking. Density functional theory (DFT) calculations further show that these coherent interfaces possess negative interfacial energies (−8.7 to −19.5 mJ m^−2^), making their formation thermodynamically favorable—an effect opposite to the positive excess energies of conventional grain and twin boundaries.

**Fig. 1:**
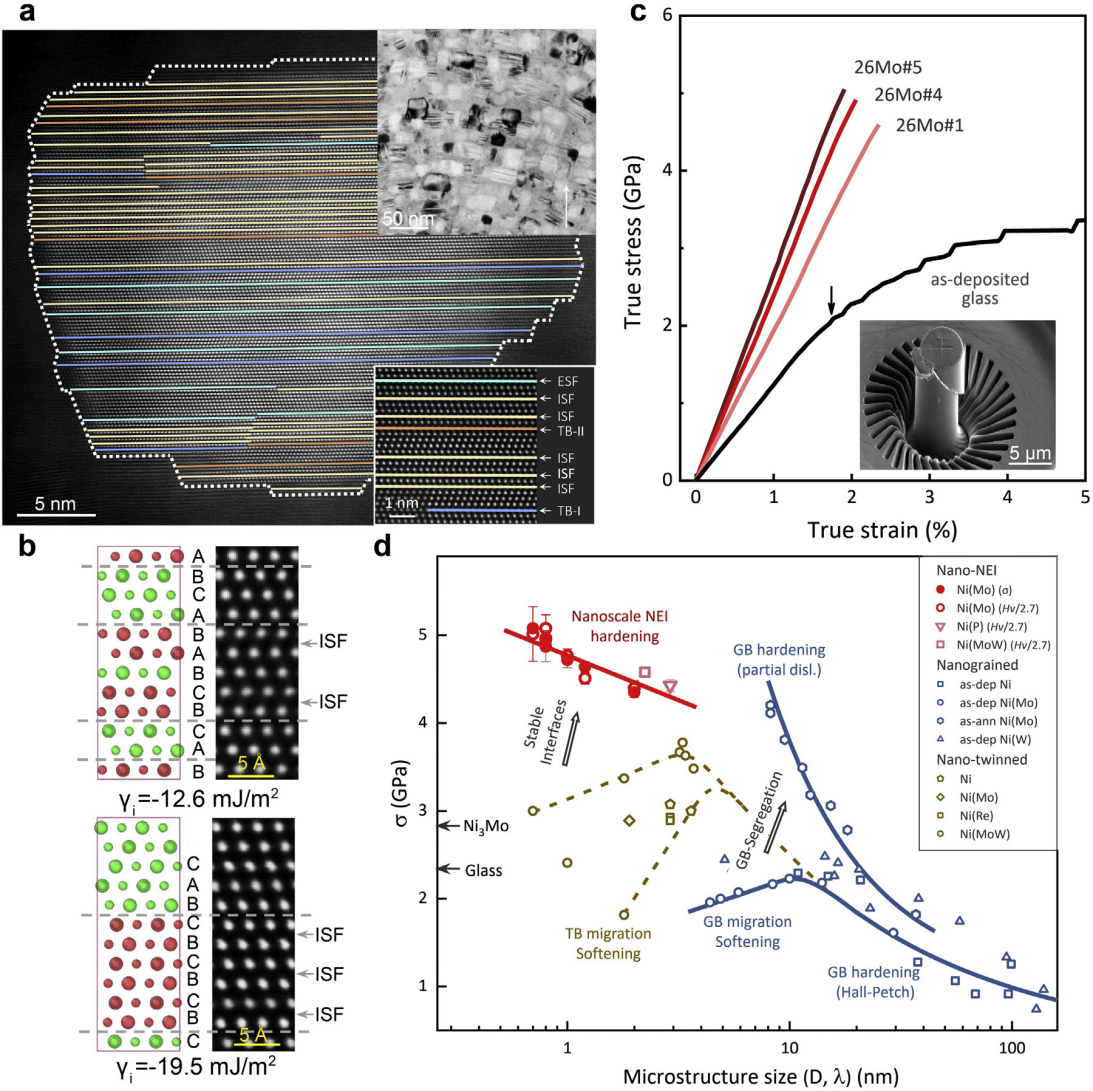
**Microstructure characterization and mechanical properties of NiMo alloy with NEIs [**5**].** (a) A HAADF-STEM image of a grain in the crystallized Ni(26 at.%Mo) alloy with TBs (type-I and type-II), intrinsic stacking faults (ISFs), and extrinsic stacking faults (ESFs). Insets are the typical bright-field transmission electron microscopy (TEM) image of crystallized NiMo alloy and a magnified HAADF-STEM image. (b) Intermixed phases of 6 ABAB layers (HCP) plus 6 ABCABC layers (FCC) separated in different ways (DFT versus high-resolution TEM images) and calculated interfacial excess energies. The small and large spheres represent Ni and Mo atoms, respectively. (c) True stress-true strain curves from micro-pillar compression tests of the as-deposited glass and the crystallized samples with different density of NEI. Inset is the fracture morphology of the sample after tests. (d) Variations of the yield strength versus microstructure size measured from micropillar compression and microhardness tests (*H*_v_/2.7) for Ni(Mo), Ni(MoW), and Ni(P) samples with nanoscale NEIs. The data of nanograined Ni-Mo alloys and nano-twinned Ni and Ni alloys from the literature are included for comparison. Solid lines represent data fitting to the corresponding samples.

These highly dense, negative-excess-energy interfaces (NEIs) are intrinsically stable and effectively impede both dislocation motion and interfacial migration. As a result, dislocation-mediated plastic deformation is fully suppressed, enabling continuous strengthening even at the sub-nanometer scale (Fig. 1c, d). The alloy consequently attains an ultrahigh strength of ∼5 GPa, exceeding half of its theoretical strength—a feat rarely achieved for metallic systems. Furthermore, the formation of NEIs markedly enhances stiffness: the elastic modulus increases linearly with interface density and reaches 254.5 GPa when the NEI fraction approaches 40%. This value not only surpasses that of metallic glass but also exceeds that of the ordered Ni₃Mo intermetallic compound. Such simultaneous enhancement of strength and stiffness represents a remarkable advance for bulk metals. Finally, DFT analyses suggest that similar NEIs also exist in other binary Ni-based alloys, including Ni–W, Ni–Ta, Ni–Nb, Ni–Mn, and Ni–V, indicating that this NEI-strengthening strategy could be broadly applicable across diverse alloy systems, including multicomponent alloys.

Previous studies have shown that constructing stable high-angle grain boundaries [6] or supra-nano dual-phase structures via solute segregation can also sustain strengthening at a size below 10 nm [7]. However, further refinement of these structures, especially into the sub-nanometer regime, has proven difficult, and continuous refinement often fails to sustain ultrahigh strength. The introduction of low-energy TBs can only extend strengthening down to structural scales of about 2 nm—the minimum attainable twin lamella thickness [[8], [9], [10]]. From a thermodynamic perspective, the emergence of NEIs aligns naturally with the formation of sub-nanometer structural dimensions. The creation of these near-limit sub-nanometer architectures and their sustained strengthening represents another milestone in the development of nanostructured metals.

In essence, tailoring energetically favorable interfaces at the atomic scale introduces a new design parameter for metals. The discovery of NEIs opens unprecedented opportunities for both structural and functional applications in metallic materials. In structural alloys, their stability against coarsening and radiation damage suggests potential use in high-temperature and radiation-resistant components, such as those in nuclear reactors and deep-space systems. Their exceptional strength makes them attractive for tribological applications, where resistance to wear and friction is critical, as well as for strong interconnects and micro-electro-mechanical systems (MEMS) that require high mechanical reliability at diminishing length scales. Beyond structural performance, the unique atomic and electronic configurations at NEIs may introduce new degrees of freedom for tuning functional properties. The altered bonding states and modified electron density at sub-nanometer interfaces could influence charge transport, magnetic exchange, and catalytic activity, providing new strategies for designing materials with tailored electrical conductivity, magnetism, or catalytic reactivity. Such interface engineering may enable high-efficiency electrocatalysts, corrosion-resistant coatings, and magnetically responsive materials.

## CRediT authorship contribution statement

**Fenghui Duan:** Writing – original draft, Investigation, Conceptualization. **Zhen Yu:** Writing – review & editing, Writing – original draft, Conceptualization. **Hanzheng Xing:** Writing – review & editing, Investigation. **Jian Lu:** Writing – review & editing, Investigation, Conceptualization.

## Declaration of competing interest

The authors declare that they have no conflicts of interest in this work.
